# Extensive Causative Esophagitis Caused by Thermal Injury: A Case Report and Review of the Literature

**DOI:** 10.1155/2017/8243567

**Published:** 2017-07-19

**Authors:** Cherng Harng Lim, Hsu-Heng Yen, Wei-Wen Su, Cherng-Jyr Lim, Hao-Chien Tsai, Shi-Ting Chen

**Affiliations:** ^1^Division of Gastroenterology and Hepatology, Department of Internal Medicine, Changhua Christian Hospital, Changhua City, Taiwan; ^2^Department of Emergency Medicine, Cathay General Hospital, Changhua City, Taiwan; ^3^Nursing Department, Changhua Christian Hospital, Changhua City, Taiwan

## Abstract

Esophagus thermal injury is a rare case that can be easily overlooked by practitioners. We herein present a case of thermally induced diffuse corrosive esophagitis with complaints of dysphagia and retrosternal chest pain after having steamed pork. A thorough disease course was demonstrated by serials of endoscopy images and video. A comprehensive review of articles and a concise overview of esophageal thermal injury clinical manifestation, disease process, typical endoscopy features, pharmacomanagement option, and outcomes will be conducted in this article.

## 1. Introduction

Acute esophagus thermal injury (ETI) is considered a reversible esophagus injury as a result of ingestion of hot foods and hot beverage or iatrogenic causes, leading to dysphagia, odynophagia, and retrosternal burning sensation. The endoscopic presentation of ETI varies and can mimic the features of chemical related corrosive injury, infection, and inflammation esophagitis which might need histopathology to be excluded. One of the distinct esophagogastroduodenoscopy (EGD) features is alternating white and reddish mucosa bands along the esophagus, resembling candy-cane appearance [1].

Herein, we report a case of diffuse corrosive esophagitis which was inflicted by steamed pork meat. Medical treatment and a series of images, videos regarding the endoscopic manifestation and healing process, were highlighted in this report. A total of 13 articles published in English literatures from 1982 to 2015 were reviewed for the purpose of analyzing the clinical manifestations, endoscopic features, histology findings, treatment options, and prognosis of ETI.

## 2. Manuscript

**Figure 1:**
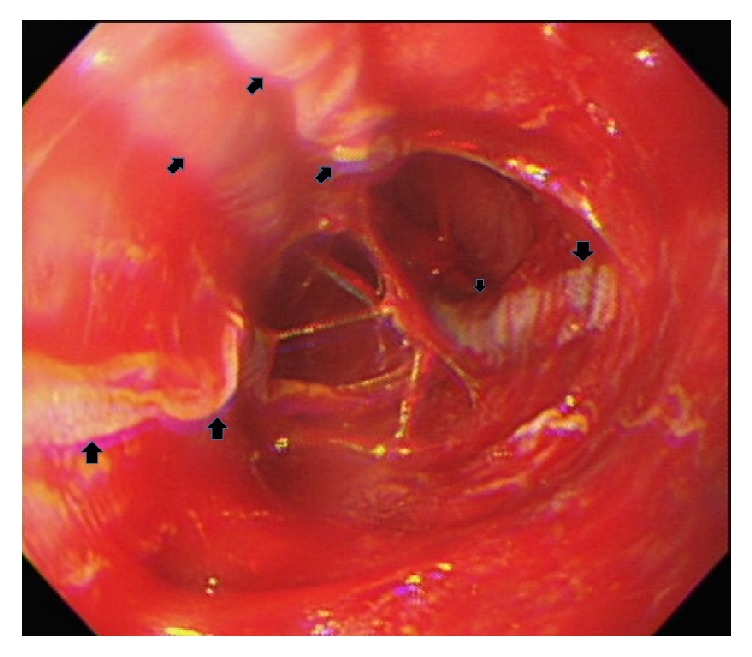
Initial endoscopy view (postthermal injury day 1). A diffuse corrosive surface with longitudinal thin, exuding, white merely scaling membranes (arrow), intervening with friable, spontaneous bleeding mucosa.

A 50-year-old gentleman presented to emergency department with progressive swallowing difficulty and retrosternal chest pain (pain score: 7/10) for about 20 hours. On arrival, his body temperature was 35.8°C, blood pressure was 155/99 mmHg, and pulse rate was 76 beats/minute. The chest radiography appeared normal and electrocardiogram showed normal sinus rhythm. Laboratory studies were within the reference ranges. There was no relevant medical history reported at that time. Esophagogastroduodenoscopy (EGD) demonstrated a diffuse mucosa erosion almost encircling the whole lumen, along with longitudinal, whitish, exuding, merely detached membranes extending from upper third to lower third of esophagus. Distal part of esophagus showed edematous, corrosive mucosa with spontaneous bleeding, without involving esophagogastric junction and gastric mucosa (Figure 1). Histopathology studies of esophagus biopsy reported ulcer with granulation tissue and inflammatory cell infiltration. There was absence of fungal pseudohyphae and viral inclusion bodies. Meanwhile, he acknowledged that he had swallowed a piece of hot steamed pork about 20 hours before. Subsequently, he felt progressive burning and pain sensation over his retrosternal area, particularly swallowing or drinking water. On the next day, his dysphagia symptom worsened making him unable to consume anything, even water. One episode of tarry stool on the day he visited the emergency department was noted. He denied consuming any caustic substances including detergents, pesticide, lye, or other chemical substances. This information was verified by his family members. In light of his medical history, endoscopy finding, and histology features, a diagnosis of corrosive esophagitis inflicted by thermal injury was made. Intravenous proton pump inhibitor (PPI, esomeprazole 80 mg/day) and sucralfate suspension (4 g/day) were prescribed to protect esophagus mucosa and avoid secondary acid reflux injury. During his hospitalization, generalized skin rash was noticed; drug allergy to esomeprazole was suspected; thus esomeprazole was changed to histamine-2 receptor antagonists (H2RA, Famotidine 40 mg/day), along with peripheral parenteral nutrition administration. Follow-up EGD on the 5th day showed alternating irregular white bands with hyperemic mucosa which bled easily on touching throughout the involved esophagus. After a week of treatment, his symptom improved gradually, cool liquid diet was tried initially, and normal diet was resumed on the 10th day of admission. He was discharged on his 11th day of admission and kept on Lansoprazole treatment for 1 month. Subsequent EGD follow-up on 10th and 30th day revealed marked improvement of mucosa. After 5 months of follow-up he was totally free of the aforementioned symptoms without any delayed complication.

## 3. Discussion

Acute ETI is a rare esophageal disease which can occur after consuming hot liquid and solid substances or iatrogenic insults. The prevalence of ETI has not yet been identified and is not easily reported [2]. It is more common in Eastern population owing to food culture. There was no direct evidence regarding coagulation abnormalities or any underlying diseases correlated with ETI. We conducted a search on PubMed and Medline Database with following terms: “(esophagus) AND (thermal injury OR burn injury OR candy-cane)”; all links and bibliography reference articles were explored and iatrogenic related ETI were excluded; a total of 18 cases were retrieved from 13 published English literatures (Tables 1, 2, and 3).

Based on our reviewed data, acute ETI caused by hot drinks and foods comprised 55% (10/18) and 40% (7/18), respectively. There was only 1 case reported in the literatures inflicted by smoking freebase cocaine. Majority of the patients, up to 72%, presented with odynophagia and/or dysphagia, 39% presented with chest discomfort, and only a small number presented with epigastric pain, dyspnea, hematemesis, melena, and so forth (Table 1). The clinical presentations often depend on the involved area. Six out of 18 of the cases with proximal esophagus involvement had the symptoms of hematemesis, dyspnea, or hoarseness.

**Figure 2:**
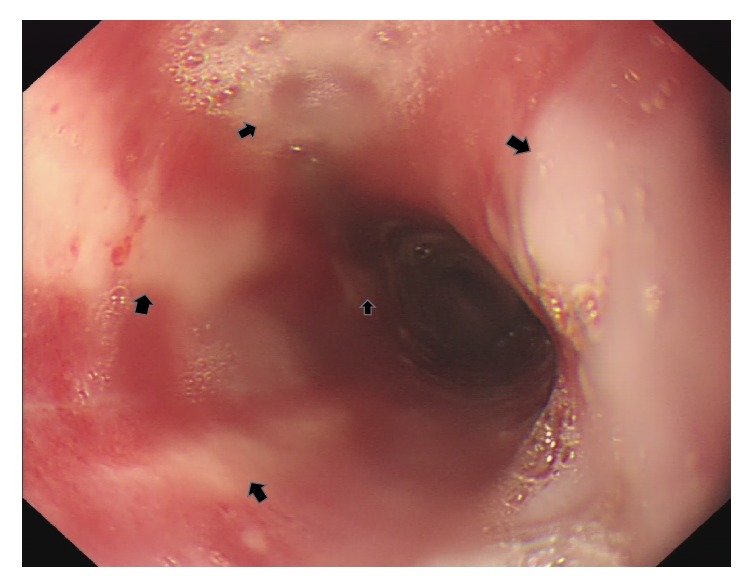
Second endoscopy view (postthermal injury day 5). Alternating geographic, longitudinal, geographic, whitish pseudomembranous (arrow) and inflamed, erythematous mucosa.

**Figure 3:**
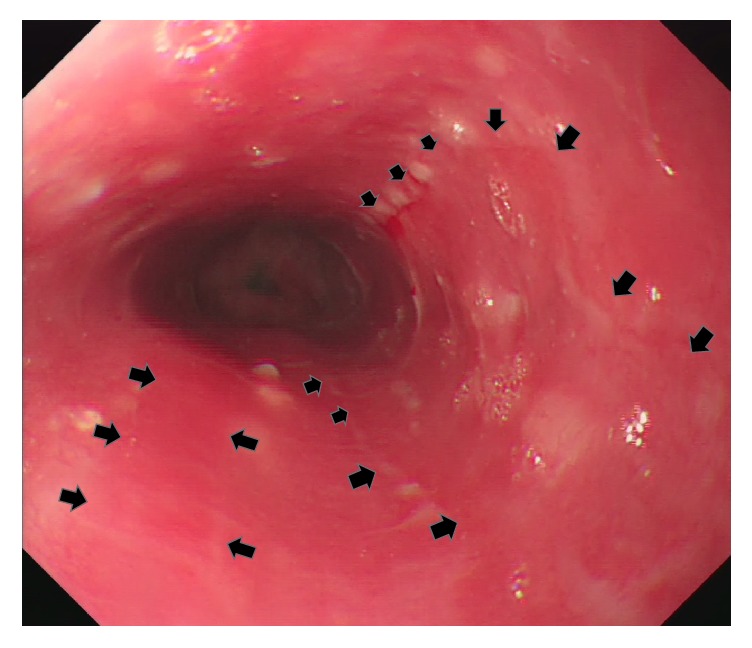
Third endoscopy view (postthermal injury day 10). Several healing hyperemic mucosa scar tracts along esophagus (arrow).

Endoscopic features of ETI vary greatly, ranging from mild erythema to blister or ulcerative lesion, as with the affected area. It can be localized, linear, or diffused which can mimic variety of esophageal disorders such as chemically induced corrosive esophagitis, infectious esophagitis, inflammation caused by radiation, and pill esophagitis. However, there is one particular endoscopic characteristic unique to ETI referred to as candy-cane esophagus [1], but this only occurs in 22% of patients. According to our reviewed data, the most common presentation was longitudinal lesion (78%), as a result of thermal tract flow along esophagus, either manifested as pseudomembranous alone (8/14), erythematous (8/14), ulcerative (4/14), or mixed mucosa (7/14) (Table 2). Through our case study, we postulated that the early stages of ETI resemble first- or second-degree (partial-thickness) skin burns, which either appear as hyperemic mucosa or whitish exuding blisters depending on the depth of injury, configured in a longitudinal pattern. Peeling or rupture of blisters may result in a longitudinal mucosa erosion or ulcer. Subsequent edematous change may attribute to temporary intraluminal stenosis. Mucosa regeneration can be observed after blister exfoliation, leaving an erythematous mucosa band. In contrast to previous article [5], our case showed that the whitish exuding blisters, which withstand from previous insult, gradually turned into pseudomembranous lesion (Figure 2). Alternation of the erythematous mucosa band and the pseudomembrane lesions formed the “candy-cane” appearance. As the erythematous mucosa bands heal they will leave scar tracts along the esophagus [7] (Figure 3). This healing process is clearly demonstrated in our case (watch the* video *in Supplementary Material available online at https://doi.org/10.1155/2017/8243567). Moreover, the endoscopic features have direct correlation with the depth and severity of thermal injury which in turn depends on the following factors: material properties (size, temperature, and form), exposure duration, and time to endoscopy. On the other hand, about 33% of the cases had evidence of oral and upper airway involvement in endoscopy, and 2 of the cases were complicated with airway obstruction [8]. Therefore, the oral cavity, laryngopharynx, and vocal cord should be investigated simultaneously during endoscopy. Interestingly, none of the cases ever reported gastric mucosa involvement directly to thermal injury. In addition, no perforation or severe blood loss events were mentioned in our collected articles.

Esophagus biopsy is not mandatory in ETI; less than half of the cases underwent biopsy via EGD (Table 2). Moreover, the histopathological findings of ETI in acute stage were unspecific, including necrosis, inflammatory cell infiltration, parakeratosis hyperplasia, and granulation tissue. Thermal involvement beyond submucosa layer was demonstrated in one case with evidence of fibrosis from submucosa to adventitia layer resulting in delayed esophagus stricture [8]. We reasonably infer that depth of thermal insult is one of the major determinants of outcome and manifestation in clinical aspect, yet solely using biopsy via EGD for histopathology evaluation is inadequate owing to its limited specimen size. The main purpose of biopsy in this aspect is to provide useful information contributing to excluding other diseases, particularly those with atypical endoscopy features.

The clinical course of ETI was considered relatively benign and reversible. Most cases were treated successfully with conservative treatment, such as avoiding further thermal insult (17%) and antisecretory treatment, such as PPIs (67%) and H2RAs (11%) or in combination with sucralfate suspension (33%) to prevent further injury from reflux gastric acid. The treatment duration in each case was inconsistent, ranging from 0 to 1 month (Table 3). There is no consensus yet as to the grading of ETI, optimum treatment options, and duration of treatment. Individualized treatments are still favored, based on depth, extending of thermal injury, and organ involvement which contribute to overall outcomes. On the other hand, airway obstruction is one deadly complication as there were two published cases that required intubation and tracheotomy to obtain airway [8]. Therefore airway evaluation and protection should be the first priority before undergoing further endoscopy examination. Steroid had been applied in one of the cases for the purpose of ameliorating trachea edema. In fact, there was no direct evidence supporting their use in thermal injury [11, 12], but exposing the risk of infection and delayed wound healing. Concomitant transient ischemic heart event [4] was reported in ETI induced by smoking freebase cocaine, albeit more likely due to cocaine itself.

The prognosis of ETI is generally favorable and is directly related to the depth and severity of thermal injury. One ETI case developed delayed esophageal stenosis requiring esophagus reconstruction [8]. Whether there is an association between acute ETI and esophageal cancer is not known. Although more studies have pointed out that “long term” consuming high-temperature beverages or food may lead to esophageal cancer as a result of impairing mucosa barrier and chronic inflammation [13], speaking of “long term” is related to amount, duration, and temperature of consumption; thus whether it can apply for those after one episode of acute thermal insult is not well described. None of the published ETI cases have ever reported to have esophageal cancer, yet the average follow-up duration was 2.4 months (range from 2 days to 12 months). Therefore, further investigation regarding the association of carcinogenesis might need substantial studies with long term follow-up to clarify.

In conclusion, ETI can easily be overlooked by practitioners due to its rarity. Often times, patients left out important clues for diagnosis during history taking. By recognizing the distinctive features of endoscopic findings in ETI, treating physician can obtain relevant history and make the correct diagnosis easily. For atypical cases, a prompt investigation of etiology is required; further biopsies might be needed to exclude other possibilities. Meanwhile, airway evaluation should be assessed by endoscopist other than gastrointestinal tract alone. The endoscopic grading system for ETI is yet to be standardized which is relevant to treatment strategies and prognosis.

## Supplementary Material

Clinical course of esophagus thermal injury.

## Figures and Tables

**Table 1:** Summary of clinical manifestation in ETI published cases.

Number	Age/gender	Material	Clinical manifestation		
			Odynophagia, dysphagia	Chest discomfort	Other
1	22/M	Microwave heated jelly roll	√		
2 [2]	21/F	Microwave heated lasagna	√	√	
3 [1]	66/M	Hot beverages	√	√	
4 [1]	72/F	Hot soups			Melena
5 [3]	20/M	Hot hamburger	√		
6 [4]	55/M	Smoking freebase cocaine			Melena, diaphoresis, hypotension
7 [5]	30/F	Hot tea	√	√	Hematemesis
8 [6]	69/M	Hot tea	√	√	Epigastric discomfort
9 [7]	53/M	Hot prawn	√	√	
10	79/M	Microwaved lasagna	√		Drooling, hoarseness
11	45/F	Hot tea	√		Hematemesis
12	52/M	Stew		√	
13	29/M	Hot water	√		
14	57/F	Hot water			Anemia
15	54/M	Hot tea	√		
16 [8]	28/M	Hot coffee	√		Dyspnea
17 [9]	47/F	Hot dumpling	√	√	
18 [10]	19/M	Hot tea			Hematemesis

M, male; F, female; N/A, not applicable.

**Table 2:** Summary of endoscopy manifestation and treatment in ETI published cases.

Number	Timeline	Image presentation		Histology
		Esophagus	Other	
		Apparatus/length/circumferences (i) Finding	Area/finding	
1	3 days	EGD/28–38 cm from incisors/partial (i) Multiple linear erythematous	N/A	N/A

2 [2]	3 days	Esophagogram/mid to distal/partial (i) Longitudinally linear collection and filling defect	N/A	N/A

3 [1]	1 month	EGD/upper to lower/whole (i) Candy-cane appearance	N/A	necrotic, anucleated, nonviable epithelium

4 [1]	3 weeks	EGD/upper to lower/whole (i) Candy-cane appearance	N/A	Necrotic anucleated nonviable epithelium

5 [3]	12 days	EGD/30 cm from incisor/partial (i) Single longitudinal ulcer	N/A	N/A

6 [4]	2 days	EGD/distal/whole (i) Candy-cane appearance	Heart/Transient cardiac ischemia;	parakeratosis, squamous hyperplasia, inflammatory cell infiltration

7 [5]	7 days	EGD/full length/partial (i) Pseudomembranous mucosa band with hyperemic mucosa	N/A	Ulcer with granulation tissue

8 [6]	1 week	EGD/full length/whole (i) Diffuse pseudomembranous mucosa and erosion	Oral cavity/whitish plaque, erosion	
	10 days	(ii) Linear plaque, lower esophagus small fibrotic changes		

9 [7]	3 days	EGD/full length/partial (i) Longitudinal ulcer band		Ulcer with activated endothelial cell
	8 days	EGD/full length/partial (i) Pseudomembranous mucosa band		

10	1 day	EGD /full length/N/A (i) Erythema and swelling mucosa	Pharyngeal, laryngeal & vocal cord/inflammation	N/A

11	7 days	EGD/hypopharynx to 25 cm from incisors/partial (i) Pseudomembranous mucosa in geographic shape	arytenoid folds/edematous mucosa	Ulceration with inflammation, atypia epithelial
	14 days	(ii) Candy-cane appearance		

12	7 days	EGD/middle to distal esophagus/partial (i) Longitudinal pseudomembrane and erosion	N/A	N/A

13	Same day	EGD/upper to middle/whole (i) Pseudomembrane and hyperemic mucosa	N/A	N/A
	7 days	(ii) Whitish fibrosis and edematous hyperemic mucosa		

14	Few days	EGD/lower esophagus/N/A (i) Friable mucosa, fibrosis, and edema	Oral cavity/pseudomembrane mucosa	N/A

15	1 day	ED/34 cm from incisor/partial (i) Focal ulcer	N/A	necrosis with white cells and cellular debris

16 [8]	N/A	EGD/N/A/N/A (i) Failed to pass through	Laryngopharyngeal/edematous mucosa	Fibrosis over submucosa and muscularis propria and adventitia
	40 days	(ii) Healing of edematous mucosa		
	5 months + 53 days	EGD/19 cm from incisor to distal/whole (i) Circumference stenosis	N/A	

17 [9]	1 day	EGD/upper to middle (30 cm from incisor)/partial/ (i) Longitudinal pseudomembrane	Pharynx/pseudomembrane mucosa	N/A

18 [10]	N/A	EGD/N/A/N/A (i) Diffuse ulcerations with candy-cane appearance	N/A	N/A

EGD, esophagogastroduodenoscopy; N/A, not applicable.

**Table 3:** Summary of treatment and follow-up duration in ETI published cases.

Number	Medication	Time to resolve	Follow-up duration
1	Avoid	Symptom resolves in 2 days	2 days
2 [2]	H2RA (Famotidine) for 1 month	Symptom improves in 1 week	N/A
3 [1]	Avoid	N/A	N/A
4 [1]	Avoid	N/A	N/A
5 [3]	PPI (omeprazole) for 1 month	Clinical improvement in 1 month	2 months
6 [4]	PPI for 1 month	N/A	12 months
7 [5]	PPI (Pantoprazole) for 4 weeks	N/A	2 months
8 [6]	PPI (Pantoprazole)	Symptom improves in 3 days	11 days
9 [7]	PPI (Pantoprazole) for 1 month	Symptom improves in 8 days	2 months
10	Steroid (dexamethasone) + intubation	Clinical improves in 2 days	2 days
11	PPI (Lansoprazole) + sucralfate	N/A	2 weeks
12	H2R A (ranitidine) + sucralfate for 3 weeks	Endoscopic improvement in 3 weeks	3 weeks
13	PPI (Lansoprazole) + sucralfate	Symptom improves in 1 week	1 week
14	PPI (Lansoprazole) + sucralfate	Endoscopic improvement in 1 week	1 week
15	PPI (Lansoprazole) + sucralfate	Endoscopic improvement in 1 week	1 month
16 [8] 17 [9]	PPI + tracheostomy	N/A	10 months
	Esophagus reconstruction	N/A	
18 [10]	PPI (Lansoprazole)	Symptom improves in 2 days	2 months
1	PPI (esomeprazole) + sucralfate	Endoscopic improvement in 1 month	1 month

H2RA, histamine-2 receptor antagonist; PPI, proton pump inhibitor; N/A, not applicable.

## References

[1] Dutta S. K., Chung K. Y., Bhagavan B. S. (1998). Thermal injury of the esophagus. *New England Journal of Medicine*.

[2] Javors B. R., Panzer D. E., Goldman I. S. (1996). Acute thermal injury of the esophagus. *Dysphagia*.

[3] Eliakim R. (1999). Thermal injury from a hamburger: a rare cause of odynophagia. *Gastrointestinal Endoscopy*.

[4] Cohen M. E., Kegel J. G. (2002). Candy cocaine esophagus. *Chest*.

[5] Choi E. K., Gin H. L., Jung H.-Y. (2005). The healing course of thermal esophageal injury: a case report. *Gastrointestinal Endoscopy*.

[6] Hoon G., Hyeon W. Y., Sung H. J. (2007). Esophageal thermal injury by hot adlay tea. *Korean Journal of Internal Medicine*.

[7] Chung W. C., Paik C. N., Jung J. H., Kim J. D., Lee K.-M., Yang J. M. (2010). Acute thermal injury of the esophagus from solid food: clinical course and endoscopic findings. *Journal of Korean Medical Science*.

[8] Kitajima T., Momose K., Lee S. (2014). Benign esophageal stricture after thermal injury treated with esophagectomy and ileocolon interposition. *World Journal of Gastroenterology*.

[9] Wu C.-H., Bair M.-J., Lin I.-T., Lee Y.-K., Chen H.-L. (2015). Early endoscopic finding of esophageal thermal injury after having spicy hot pot. *Advances in Digestive Medicine*.

[10] AC A., Rajma J. (2016). Candy cane appearance of the esophagus caused by acute thermal injury. *Clinical Gastroenterology and Hepatology*.

[11] Cha S. I., Kim C. H., Lee J. H. (2007). Isolated smoke inhalation injuries: Acute respiratory dysfunction, clinical outcomes, and short-term evolution of pulmonary functions with the effects of steroids. *Burns*.

[12] Herndon D. N., Barrow R. E., Linares H. A. (1988). Inhalation injury in burned patients: effects and treatment. *Burns*.

[13] Islami F., Boffetta P., Ren J.-S., Pedoeim L., Khatib D., Kamangar F. (2009). High-temperature beverages and foods and esophageal cancer risk—a systematic review. *International Journal of Cancer*.

